# Age-related inflammation and insulin resistance: a review of their intricate interdependency

**DOI:** 10.1007/s12272-014-0474-6

**Published:** 2014-09-20

**Authors:** Min Hi Park, Dae Hyun Kim, Eun Kyeong Lee, Nam Deuk Kim, Dong Soon Im, Jaewon Lee, Byung Pal Yu, Hae Young Chung

**Affiliations:** 1 Molecular Inflammation Research Center for Aging Intervention (MRCA), College of Pharmacy, Pusan National University, Busan, 609-735, Republic of Korea; 2 Department of Physiology, The University of Texas Health Science Center at San Antonio, San Antonio, TX 78229-3900, USA

**Keywords:** Insulin resistance, Molecular inflammation, Aging

## Abstract

Chronic inflammation is a major risk factor underlying aging and the associated diseases of aging; of particular interest is insulin resistance during aging. Chronic inflammation impairs normal lipid accumulation, adipose tissue function, mitochondrial function, and causes endoplasmic reticulum (ER) stress, which lead to insulin resistance. However, some studies show that insulin resistance itself amplifies chronic inflammation. The activity of the insulin-dependent Akt signaling pathway is highlighted because of its decrease in insulin-sensitive organs, like liver and muscle, which may underlie insulin resistance and hyperinsulinemia, and its increased levels in non-metabolic organs, such as kidney and aorta. In that the prevalence of obesity has increased substantially for all age groups in recent years, our review summarizes the data showing the involvement of chronic inflammation in obesity-induced insulin resistance, which perpetuates reciprocal interactions between the chronic inflammatory process and increased adiposity, thereby accelerating the aging process.

## Introduction

Chronic, low-grade, systemic inflammation is wildly accepted as a significant risk factor underlying aging and major diseases (Yu and Chung 2006). Inflammation, as part of the key innate immune defense system, is an essential protective mechanism that fights against not only invading pathogens and other exogenous harmful materials, but also endogenously produced substances (Mogensen 2009) that are tightly regulated under normal conditions. However, unresolved chronic inflammation is known to exacerbate and underlie age-related diseases such as cancer, type 2 diabetes, cardiovascular diseases, arthritis, osteoporosis, and Alzheimer’s disease (Howcroft et al. 2013).

Strong evidence was presented on a plausible relationship between inflammation and obesity-related diseases (Gregor and Hotamisligil 2011). It was shown that an age-related increase in visceral adiposity results in high circulating levels of pro-inflammatory cytokines that interfere with insulin signaling during the aging process (Sepe et al. 2011). Pro-inflammatory macrophages, a major source of cytokines, are present in much higher numbers in the adipose tissue of obese subjects than lean subjects (Heilbronn and Campbell 2008).

Pro-inflammatory cytokines were shown to act in an autocrine or paracrine manner to induce insulin resistance in peripheral tissues and macrophages (Schenk et al. 2008; Tilg and Moschen 2008). The effect of pro-inflammatory cytokines that induces insulin resistance begins by the ameliorated tyrosine phosphorylation of insulin receptor substrate (IRS)-1.

In this review, we summarize recent work on the close association between chronic inflammation and insulin resistance during aging process.

## Molecular insights into the inflammatory process during aging

**Fig. 1:**
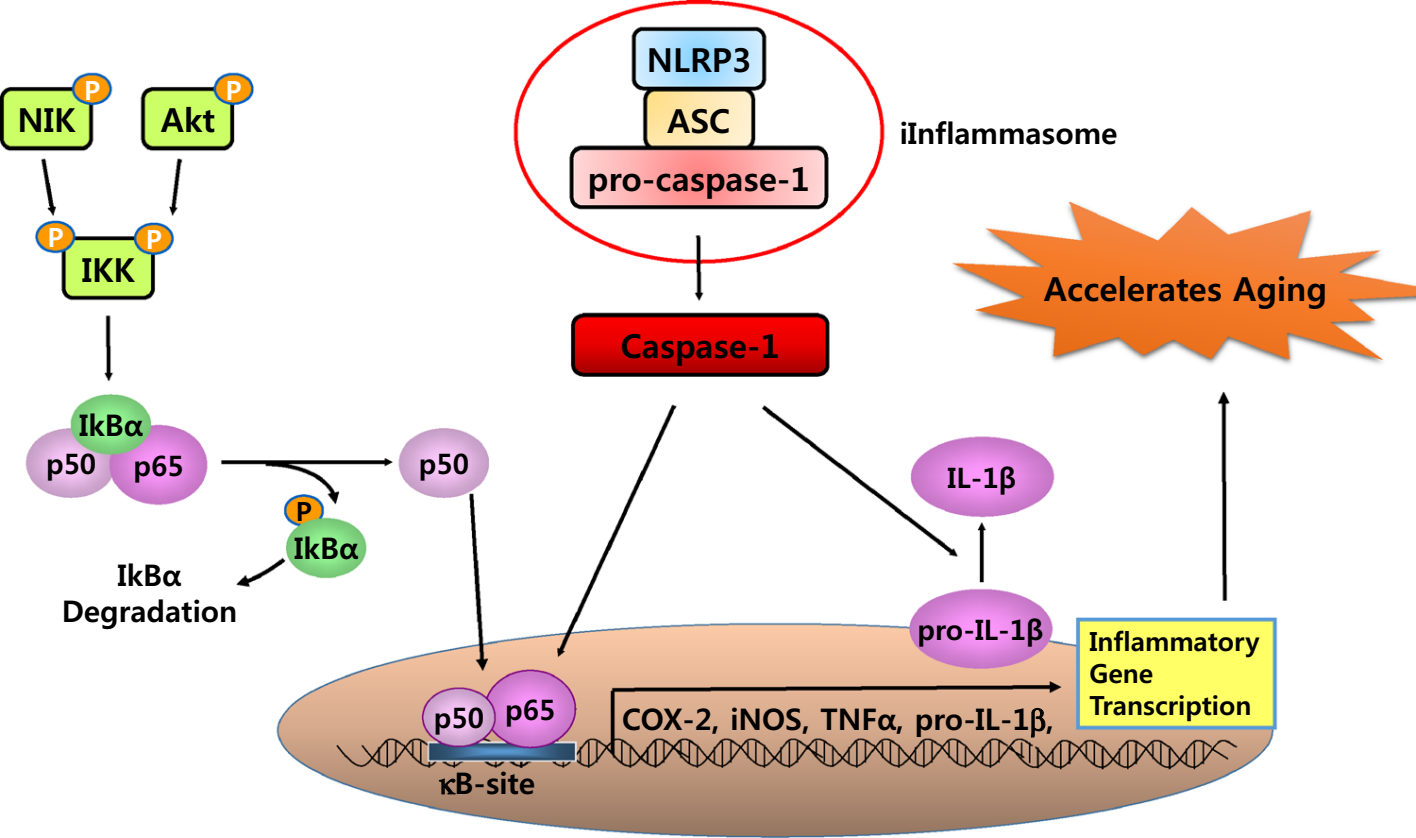
Signaling pathways downstream of NIK and Akt activation. Extracellular receptors bind to their ligands and signal via NF-κB inducing kinase (NIK) or Akt molecules. This leads to phosphorylation of the IκB kinase (IKK) subunits and the subsequent phosphorylation of IκBα, which leads to its ubiquitination and proteasomal degradation. Inflammasome activation also triggers signaling through nuclear factor-κB (NF-κB) and Interleukin-1β (IL-1β). NF-κB is then released into the nucleus where it acts as a transcription factor and stimulates inflammatory responses

In chronic inflammation, the inflammatory response is driven by the versatile transcription factor, nuclear factor-κB (NF-κB) during aging. NF-κB is activated by the IκB kinase (IKK)/NF-κB inducing kinase (NIK) and the mitogen activated protein kinase (MAPK) pathways (Fig. 1). Once activated, NF-κB promotes the expression of several other pro-inflammatory cellular mediators, like prostaglandins (PGs), inducible nitric oxide synthase (iNOS), and inflammation-related cytokines. PG metabolites are produced by the action of the enzyme, cyclooxygenase (COX). These are important pro-inflammatory mediators that are known to increase reactive oxygen species (ROS) production, which leads to DNA and tissue damage (Yu and Chung 2001). PGs-induced ROS generation steadily increases in peripheral tissue during aging (Chung et al. 2011). Several studies using COX-2 deficient mice reveal impaired inflammatory responses through the attenuation of PGs generation (Morham et al. 1995; Dinchuk et al. 1995). Not only ROS but also pro-inflammatory cytokines such as interleukin (IL)-1β and IL-6 are enhanced by PGs (Aoki and Narumiya 2012). Nitric oxide (NO) is one of the most physiologically important radical species known to induce the chronic inflammatory response. NO is synthesized by iNOS and can cause pathogenesis and destruction of various tissues, similar to that seen in chronic inflammatory (Zamora et al. 2000).

The recently established inflammasome, an essential component of the innate immune response, plays a key role in the inflammatory process by the secretion of the cytokines, IL-1β and IL-18 (Agostini et al. 2004). The close cooperative actions between the inflammasome and NF-κB undoubtedly exacerbates the chronic inflammatory status during the aging process (Howcroft et al. 2013). The most studied NLRP3 inflammasome is an intracellular protein complex composed of NOD-like receptor (NLR) proteins, apoptosis-associated speck-like protein containing a CARD (ASC), and pro-caspase-1, which result in caspase-1 activation. The formation of the inflammasome induces auto-processing and activation of caspase-1 leading to the processing of cellular substrates, namely IL-1β and IL-18. Activation of the well-studied NLRP3 inflammasome has a diverse sensing ability reported to participate in aging-related inflammatory processes. It is worth noting the recent important report by Youm et al in which the authors describe that the suppression of the NLRP3 inflammasome extended the healthy lifespan of mice by attenuating age-related degenerative changes, including cognitive decline. Based on their findings, the authors proposed that suppression of aberrant NLRP3 activity during aging may well attenuate age-related diseases by reducing chronic inflammation (Youm et al. 2013).

## Insulin resistance and altered PI3K/Akt signaling pathway

Through mechanistic exploration, numerous studies have produced various interesting data, but to date, there seems to be no unifying consensus, implying the complex nature of the subject (Muoio and Newgard 2008; Kahn et al. 2006; Saini 2010). One interesting observations is that insulin resistance, at cellular and molecular levels, is characterized by dysfunction in the IRS/phosphoinositide 3-kinase (PI3K)/Akt signaling pathway, which results in impaired glucose uptake in hepatocytes and adipocytes. The process involved in glucose uptake has a series of sequential events beginning at the insulin receptor (IR), which is a heterotetramer consisting of two α subunits and two β subunits that are linked by disulfide bonds. Insulin binds to the α subunit of the IR and activates the tyrosine kinase in the β subunits to initiate insulin signaling. Once the tyrosine kinase of the IR is activated, it promotes auto-phosphorylation of the β subunit, where phosphorylation of tyrosine residues and a conformational change are required to amplify the kinase activity. The activated receptor then phosphorylates adaptor molecules, such as IRS-1 and IRS-2, which permit the recruitment and activation of phosphatidylinositol 3-kinase (PI3-kinase). Activated PI3-kinase produces 3-phosphoinositides, phosphatidyl-inositol-3,4-bisphosphate (PIP2) and phosphatidyl-inositil-3,4,5-trisphosphate (PIP3) that bind to phosphoinositide-dependent kinase 1 (PDK1). Akt activates glucose transporter 4 (GLUT4), which moves to the cell surface to transport glucose into the cell.

## Possible causes underlying insulin resistance through inflammation activation during aging

### Lipid accumulation

Lipids have a wide variety of roles in biological systems. These roles are a consequence of their chemical and physical properties. However, excess lipid accumulation induces insulin resistance during aging. Recently studies have shown that inflammatory responses disrupt normal lipid accumulation. For example, pro-inflammatory cytokines such as IL-6 and tumor necrosis factor-α (TNFα) impair the lipid accumulation by promoting WNT signaling (Gustafson and Smith 2006). Indeed, inflammation leads to lipid accumulation through disruption of the expression of proteins related to lipid metabolism, such as sterol regulatory element binding protein (SREBP), a basic helix-loop-helix transcription factor that controls the expression of genes required for cholesterol, fatty acids, triglycerides and phospholipids synthesis (Ferre and Foufelle 2007).

A recent cDNA microarray study from our laboratory (Park et al. 2013) documented that the genes upregulated during aging contain several major inflammatory genes. The upregulated genes included NF-κB1alpha and a 90 kDa ribosomal protein, S6 kinase, which is related to mammalian target of rapamycin (mTOR) in aged rats (Park et al. 2013). In the aging transcriptome, genes that are related to both the immune response and to inflammation were upregulated with age, particularly those related to cytokine–cytokine receptor interaction, natural killer cell mediated cytotoxicity, and primary immunodeficiency (Anderson et al. 2010). Down-regulated genes, such as peroxisome proliferator-activated receptors (PPARs) associated with lipid metabolism, were also observed with aging according to the gene ontology data (Hong et al. 2010). Similarly, energy metabolism, insulin signaling, and PPAR signaling genes were down-regulated, as shown by the analysis of the protein–protein interaction network that was composed of genes differentially expressed during aging (Park et al. 2013). The involvement of inflammation was also shown by the reduction of ABCA1-mediated cholesterol efflux that was regulated by PPARs in acceleration of foam cell formation through disrupting LDL receptors-mediated negative feedback regulation in vascular smooth muscle cells and mesangial cells (Ma et al. 2008). These data further support that metabolic disorders associated with lipid accumulation are closely related to chronic inflammation at the molecular level during aging.

### Dysregulated adipose tissue

**Fig. 2:**
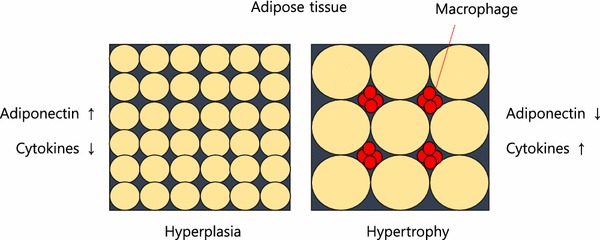
Obesity is determined by increasing both, the size and number of adipocytes. Adipogenesis can lead to a large number of new adipocytes (hyperplasia) which produce more adiponectin and less inflammatory adipokines. Conversely, hypertrophied adipocytes produce less adiponectin and more inflammatory adipokines. The prevalence of hypertrophied adipocytes in adipose tissue leads to a reduction in blood flow with subsequent hypoxia and macrophage infiltration. In addition, cytokines produced by macrophages inhibit adipogenesis

White adipose tissue (WAT) not only is involved in energy storage in the body, but also secretes a variety of bioactive molecules including adiponectin, IL-1β, IL-6, and TNFα, which enable the adipose tissue to communicate with other tissues and organs, such as the liver, skeletal muscle, and central nervous system. Through this interaction network, WAT participates in regulating important biological processes, such as food intake, energy homeostasis, and insulin resistance. Adipose tissue is at the nexus of mechanisms and signaling pathways involved in metabolic disorder, inflammation, aging and age-related diseases. Obesity and aging are affected by increasing both the size and number of adipocytes. Adipogenesis can lead to a large number of new adipocytes (i.e., hyperplasia), which produce more adiponectin and fewer pro-inflammatory adipokines. In contrast, the prevalence of hypertrophied adipocytes in adipose tissue encourages increased macrophage infiltration (Despres 2006; Goossens 2008). The recruitment of macrophages into adipose tissue is the initial event leading to obesity-induced inflammation and insulin resistance (Fig. 2).

In general, over-nutrition and aging cause adipocytes to secrete chemokines, such as pro-inflammatory monocyte chemotactic protein-1 (MCP-1) and others that provide a chemotactic gradient that attracts monocytes to the adipose tissue where they become adipose tissue macrophages (ATMs). Once pro-inflammatory ATMs migrate into adipose tissue, they also secrete their own chemokines, which attract additional macrophages creating a feed-forward inflammatory process (Olefsky and Glass 2010). Thus, ATMs perpetuate inflammation, by inducing local and systemic increases of pro-inflammatory molecules like IFNγ, IL-1β, IL-6, and TNFα, while decreasing other adipokines, such as adiponectin.

Hypertrophied adipocytes, which are up to 150–200 μm in diameter are larger than the normal distance that O_2_ can diffuse, which is 100–200 μm (Skurk et al. 2007; Brahimi-Horn and Pouyssegur 2007), which leads to a hypoxic condition. Hypoxia in obese adipose tissue has been well characterized by many researchers, and so has the induction of a key regulator, hypoxia-inducible factor-1 (HIF-1) (Hosogai et al. 2007; Wang 2007). HIF-1 is a transcription factor that accumulates during hypoxia and increases the mRNA expression of a wide variety of genes that stimulate erythropoiesis, angiogenesis, and glycolysis (Semenza 2000). Thus, it provides a major link to the pathogenesis of insulin resistance (Olefsky and Glass 2010).

Visceral adipose tissue refers to intra-abdominal fat around the intestines and it correlates with liver fat. Visceral adipose tissue has metabolic characteristics that differ from those of subcutaneous fat. It is more metabolically active with a high free fatty acid (FFA) turnover, and the increased flux of FFAs promotes insulin resistance at a cellular level and increases hepatic very low density lipoprotein (VLDL) production. Indeed, excess visceral adiposity has been shown to accompany chronic low-grade inflammation (Giorgino et al. 2005). Studies on the visceral adipose tissue from lean or obese subjects demonstrate that macrophage specific markers and chemokines involved in monocyte chemotaxis, as well as their receptors, are elevated in obese visceral fat compared with lean controls (Huber et al. 2008).

### Mitochondrial dysfunction

For many years, it has been known that mitochondrial dysfunction is associated with type 2 diabetes mellitus (T2DM) and age-related insulin resistance (Stump et al. 2003; Petersen et al. 2003). The mitochondrial oxidative phosphorylation capacity is defined as the maximal ADP-stimulated oxidative phosphorylation elicited by a high ADP:ATP ratio that reflects the maximal energy demands of the cell at saturating concentrations of substrates (Chance et al. 2006; Prompers et al. 2006). Mitochondrial oxidative phosphorylation and unlimited oxygen supply is very important for the function of the cell. However, subjects with T2DM, often have mitochondrial dysfunctions such as reduced insulin-stimulated ATP production, lower protein expression of respiratory chain subunits, decreased mitochondrial DNA, and decreased mitochondrial size and density (Ritov et al. 2005; Heilbronn et al. 2007; Szendroedi et al. 2007; Hwang et al. 2010).

In addition, several studies have shown that the oxidative phosphorylation capacity is impaired in muscle biopsy samples from patients with T2DM compared with healthy individuals (Phielix et al. 2008; Boushel et al. 2007). Moreover, the expression of genes for mitochondrial oxidative phosphorylation is related to insulin resistance (Petersen et al. 2003). Mitochondrial dysfunction results in the accumulation of fatty acid metabolites, diacylglycerol (DAG), and long chain fatty acyl-CoA (Itani et al. 2002) causes decreased fatty acid oxidation. Intracellular accumulation of DAG activates protein kinase C (PKC), which in turn activates IKK and JNK leading to increased serine phosphorylation of IRS, and consequently attenuation of insulin signaling (Griffin et al. 1999; Yu et al. 2002). Indeed, with aging, mutations and deletions occur in mtDNA, leading to impaired function of the respiratory chain and enhanced ROS production (Chomyn and Attardi 2003). Thus, mitochondrial dysfunction is highly likely one of the major causes of the age-related chronic inflammation.

### ER stress

Endoplasmic reticulum (ER) is an organelle that is comprised of a reticular membranous network that extends throughout the cytoplasm and is contiguous with the nuclear envelope. This organelle is involved in several important cellular functions that affect the homeostasis of the whole-organism. The ER is also the cellular site where all secretory and integral membrane proteins are folded and post-translationally modified in an ATP dependent process mediated by chaperones, which allows the cell to respond to misfolded proteins within the ER. These pathways are collectively known as the unfolded protein response (UPR) and are important for normal cellular homeostasis and organismal development, and may play key roles in the pathogenesis of many diseases. In addition, the ER is also a site of Ca^2+^ storage and biosynthesis for steroids, cholesterol, and lipids (van Meer et al. 2008; Ron and Walter 2007).

Recently studies demonstrated that fatty acid overload rapidly induces ER stress in pancreatic β cells and hepatocytes that leads to impaired insulin secretion and lucose uptake, respectively (Borradaile et al. 2006; Kharroubi et al. 2004). Recent studies also suggest that ER stress plays an important role in the onset of obesity, insulin resistance, and T2DM (Wellen and Hotamisligil 2005; Eizirik et al. 2008; Ozawa et al. 2005). ER stress in hepatocytes and adipocytes contributes to insulin resistance, in part through IRE1-dependent, JNK mediated inhibition of IRS-1 tyrosine phosphorylation, and increased serine phosphorylation (Ozawa et al. 2005; Hirosumi et al. 2002). Several chaperones and folding enzymes such as glucose regulated proteins 78 (GRP78; which is also known as immunoglobulin binding protein [BiP]), protein disulfide isomerase (PDI), calnexin, and calreticulin participate in protein folding in the ER. These chaperones and foldases decrease with age (Naidoo et al. 2005; Nuss et al. 2008; Rabek et al. 2003).

ER stress has been demonstrated to trigger activation of JNK as well as IKK by increasing IRE1 and leads to inducing NF-κB activation (Brown et al. 2008). Increased JNK and NF-κB signaling would then induce the expression of pro-inflammatory cytokines (Hirosumi et al. 2002). ER stress is also a major source of ROS generation due to Ca^2+^release. This increases the concentration of cytosolic Ca^2+^ and subsequently stimulates mitochondria metabolism to produces more ROS. Thus, the ER may be a proximal site that senses over-nutrition and translates it into metabolic and age-related inflammatory responses.

## Interdependency between inflammation and insulin resistance during aging

Although many studies have focused on the effects of inflammatory responses on insulin resistance, only few studies have specifically investigated the effects of insulin resistance on inflammatory responses. Exposure to excessive amounts of nutrients and energy can impair the insulin signaling in metabolically important tissues such as the liver, adipose tissue, and skeletal muscle. Insulin resistance is therefore closely associated with chronic inflammation, likely due to increased levels of pro-inflammatory cytokines.

**Fig. 3:**
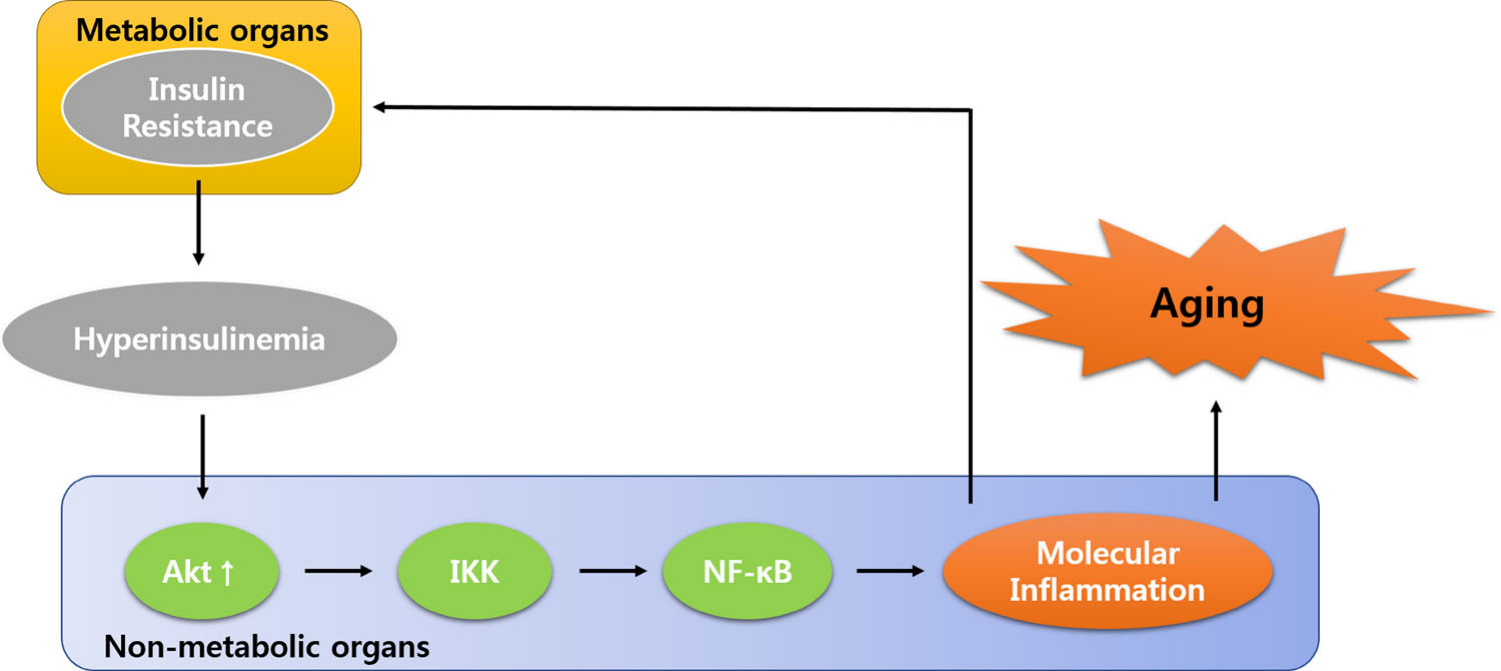
Crosstalk between insulin resistance in metabolic organs and molecular inflammation in non-metabolic organs. The serum insulin concentration is up regulated in an insulin resistance state. Insulin resistance-induced hyperinsulinemia stimulates inflammatory responses in non-metabolic organs such as kidney by activating the Akt/IκB kinase (IKK) signaling pathway

In fact, type 2 diabetes induced by insulin resistance has recently been suggested as a cause of accelerated aging (Mokdad et al. 2001). In an insulin resistant state, serum insulin levels are increased to a hyperinsulinemia state that activates the Akt/IKK signaling pathway, and thus activates NF-κB, a core transcription factor involved in the inflammatory response in non-metabolic organs (Fig. 3). Over recent years, the incidence of NF-κB activation in human kidney has risen dramatically, primarily because of the increasing prevalence of both obesity and insulin resistance (El-Atat et al. 2004). Obesity and insulin resistance both make substantial contributions to renal disease by up-regulating Akt activity. Many studies suggest that chronic insulin resistance, accompanied by hyperinsulinemia, contributes to several pathologies, such as atherosclerosis, through augmenting the effects of pro-inflammatory cytokines (Lele 2007; Goldschmidt-Clermont et al. 2005). In practice, high levels of the pro-inflammatory cytokines have been found in the adipose tissue of a diabetic rat model. They activate JNK and IKK/NF-κB signaling pathways, resulting in or from the up-regulation of potential mediators of chronic inflammation, which perpetuates reciprocal interactions between the chronic inflammation and adiposity, leading to accelerated aging.

Therefore, it is possible that therapeutic drugs for insulin resistance, such as AMPK activator or PPARα/γ dual agonist, have the potential to become anti-aging agents by ameliorating insulin resistance. Actually, metformin, an AMPK activator, extends lifespan in *C. elegans*and mice (Cabreiro et al. 2013; Martin-Montalvo et al. 2013). Indeed, it has been shown that insulin-induced FOXO1 is a master protein that turns on the expression of another key inflammatory cytokine, IL-1β that also inhibits insulin signaling (Su et al. 2009). Thus, FOXO1 inhibitors may have the potential to develop into anti-aging agents through their ability to inhibit IL-1β production.

It is still unclear whether insulin resistance or inflammation occurs first. It is clear however, that there is a vicious cycle between insulin resistance and inflammation that occurs during aging and contributes to accelerated aging. Also, over-nutrition intake, such as with a western diet, could accelerate aging by increasing both inflammation and insulin resistance.

## Conclusion

Many aging hypotheses have been proposed over the past several decades, including the recent molecular inflammation hypothesis of aging. This hypothesis of molecular inflammation is based on activities of the key NF-κB signaling pathway, which plays a crucial role in activating the systemic inflammatory response during the aging process when there is increased adiposity. Chronic inflammation due to obesity-induced ectopic tissue, such as liver and muscle lipid accumulation further exacerbates insulin resistance. A state of chronic inflammation perpetuates an insulin resistant state, and the interactions between a chronic inflammatory response and increased adiposity likely accelerate the aging process.
